# Feasibility study on dosimetry verification of volumetric‐modulated arc therapy‐based total marrow irradiation

**DOI:** 10.1120/jacmp.v14i2.3852

**Published:** 2013-03-04

**Authors:** Yun Liang, Gwe‐Ya Kim, Todd Pawlicki, Arno J. Mundt, Loren K. Mell

**Affiliations:** ^1^ Department of Radiation Oncology University of California San Diego La Jolla CA; ^2^ Center for Advanced Radiotherapy Technologies University of California San Diego La Jolla CA; ^3^ Division of Radiological Physics University of California San Diego La Jolla CA; ^4^ Department of Radiation Oncology Allegheny General Hospital Pittsburgh PA USA

**Keywords:** total body irradiation, total marrow irradiation, volumetric‐modulated arc therapy, gamma evaluation, volumetric portal dosimetry

## Abstract

The purpose of this study was to develop dosimetry verification procedures for volumetric‐modulated arc therapy (VMAT)‐based total marrow irradiation (TMI). The VMAT based TMI plans were generated for three patients: one child and two adults. The planning target volume (PTV) was defined as bony skeleton, from head to mid‐femur, with a 3 mm margin. The plan strategy similar to published studies was adopted. The PTV was divided into head and neck, chest, and pelvic regions, with separate plans each of which is composed of 2–3 arcs/fields. Multiple isocenters were evenly distributed along the patient's axial direction. The focus of this study is to establish a dosimetry quality assurance procedure involving both two‐dimensional (2D) and three‐dimensional (3D) volumetric verifications, which is desirable for a large PTV treated with multiple isocenters. The 2D dose verification was performed with film for gamma evaluation and absolute point dose was measured with ion chamber, with attention to the junction between neighboring plans regarding hot/cold spots. The 3D volumetric dose verification used commercial dose reconstruction software to reconstruct dose from electronic portal imaging devices (EPID) images. The gamma evaluation criteria in both 2D and 3D verification were 5% absolute point dose difference and 3 mm of distance to agreement. With film dosimetry, the overall average gamma passing rate was 98.2% and absolute dose difference was 3.9% in junction areas among the test patients; with volumetric portal dosimetry, the corresponding numbers were 90.7% and 2.4%. A dosimetry verification procedure involving both 2D and 3D was developed for VMAT‐based TMI. The initial results are encouraging and warrant further investigation in clinical trials.

PACS number: 87.55.Qr

## I. INTRODUCTION

Total body irradiation (TBI) is commonly used in conjunction with chemotherapy as a conditioning regimen prior to bone marrow transplantation in patients with hematological malignancies. The primary purpose of TBI is to eradicate malignant cells and cause immunosuppression to allow for engraftment of donor bone marrow. Compared to conditioning regimens based on chemotherapy alone, TBI is advantageous because it is independent of factors such as blood supply, drug activation, metabolism, or hepatic or renal function. TBI also treats chemotherapy sanctuary sites in the central nervous system and gonads.^(^ ^1^ ^)^

Conventional TBI delivery techniques include the use of anterior–posterior and posterior–anterior (AP/PA), or parallel‐opposed lateral fields, with extended source‐to‐surface distance to encompass the patient completely in the field.^(^ ^2^ ^)^ With conventional techniques, it is difficult to achieve dose uniformity within the target due to heterogeneities resulting from variations in patient thickness and tissue density. No attempt is made to spare multiple organs at risk (OARs) such as the eyes, heart, liver, and kidney. To reduce lung dose, lung blocks are often used in conventional TBI. The accurate positioning of those blocks can be challenging. Moreover, TBI is constrained by toxicities to OARs, which limits opportunity for dose escalation. Clinical trials have found that increased TBI dose significantly reduced the probability of relapse, but did not improve survival because of increased mortality from causes other than relapse.^(^ ^3^ ^)^ Conformal TBI techniques could reduce dose to OARs and permit dose escalation. This may be particularly advantageous in pediatric cases due to concern regarding developmental abnormalities and late radiation effects associated with conventional TBI.^(^ ^4^ ^)^

Intensity‐modulated radiation therapy (IMRT)‐based total marrow irradiation (TMI) is one solution to optimize treatment and permit dose escalation. Initial studies using Helical TomoTherapy (HT)‐based intensity‐modulated total marrow irradiation (IM‐TMI), total lymphatic irradiation (TLI) and total marrow and lymphoid irradiation (TMLI) have found favorable OAR dose reductions and clinical outcomes.^(^ ^5^ ^,^ ^6^ ^)^ Recent planning studies have also indicated that linac‐based fixed‐field IMRT TMI provides adequate target coverage and dose reduction to OARs compared to conventional TBI.^(^ ^7^ ^,^ ^8^ ^)^ However, the treatment duration is long and requires thousands of MUs, adversely impacting treatment accuracy.

Volumetric‐modulated arc therapy (VMAT) is a treatment planning and delivery platform using a rotating gantry and continuously changing beam aperture to produce highly conformal dose distributions with improved delivery efficiency.^(^ ^9^ ^)^ A major advantage is the ability to deliver treatment rapidly, potentially improving treatment accuracy. Recently, planning studies have found excellent target dose coverage and OAR sparing with VMAT‐based TMI.^(^ ^10^ ^–^ ^12^ ^)^ Presently linac‐based TMI studies have been limited by the lack of a dose verification procedure in the aspects of high‐resolution two‐dimensional (2D) dose verification, as well as three‐dimensional (3D) volumetric dose verification. In VMAT, verifying the whole plan while the gantry is rotating is challenging. It demands a high accuracy of quality assurance (QA) on the actual beam delivery. There have been strategies for performing 3D total plan verification based on the actual dose measured with electronic portal image device (EPID) images in combination with 3D dose reconstruction algorithms that relate the measured portal images to the dose delivered inside the phantom or patient.^(^ ^13^ ^)^ Our institution has developed this strategy for performing verification on the VMAT‐based radiation treatment plans for cancers such as prostate, brain, and head and neck (H&N),^(^ ^14^ ^)^ but studies on volumetric dosimetry QA with VMAT‐based TMI with high resolution or in dimension are lacking.^(^ ^12^ ^,^ ^15^ ^)^

The objective of this study is to develop dosimetry quality assurance procedure involving both 2D and 3D volumetric verifications, which is desirable for a large PTV in the case of TMI. We generated TMI plans for three patients aged 9, 34, and 50 years treated with conventional TBI at our institution. During the simulation, the patient was positioned straight and flat on CT table with no positioning boards or Vac‐Lok (Med‐Tec, Orange City, IA). Subjected to the preference of the attending physicians, one patient was scanned with the arms down; the other two patients were scanned with arms up. In all the cases, the clinical target volume (CTV) was defined as the bony skeleton, from head to the mid‐femur. The mandible, maxillary bones, and forearms were excluded. The CTV was expanded with 3 mm margin to generate the PTV. The OARs were brain, eyes, lungs, heart, kidneys, and liver. For the patients with arms up during CT simulation, saline material was added surrounding H&N region for the treatment planning to avoid hot spots on the skin surface. The plans were done with a Varian Eclipse treatment planning system (Version 8.9, a DELL T5400 workstation with Windows XP32 and 4GB of RAM) (Varian Medical Systems, Palo Alto, CA) with RapidArc ability (Varian Medical Systems). The plan prescription was 12 Gy in 6 fractions. The energy of 6 MV and dose rate of 600 MU/min was used.

VMAT‐based planning involves some technical challenges due to the modulation size limitation with RapidArc: 16 cm×40 cm, which consequently requires planning with multiple isocenters. In this study we adopted the same strategy from previous studies,^(^ ^7^ ^)^ and divided the PTV into H&N, chest, and pelvic regions with separate plans. Within each plan, there were 2–3 arcs/fields whose isocenters kept the same in the left–right (LR) and anterior–posterior (AP) directions while being moved along the patient's axial direction with a separation of 12 cm. The separations of the arcs between the neighboring plans were also kept at 12 cm. The arcs were alternated clockwise and counterclockwise inter‐ and intraplan. For each patient, the chest plan was initially optimized and subsequently used as the base plan for both H&N and pelvic plan optimization, which helped to minimize the hot and cold spots at the junction area.

The target planning constraints were: 1) >90% of the PTV receives >100% of the prescription dose, 2) <20% of the PTV receives >110% of the prescription dose, and 3) normal tissue planning objectives were evaluated during optimization to ensure the dose was as low as reasonable possible. Plans were reviewed by the radiation oncologist to ensure they met the planning objectives. Since there are no OARs below the mid‐femur, AP/PA techniques can be used to irradiate the areas below the mid‐femur, as reported elsewhere.^(^ ^5^ ^)^

## II. MATERIALS AND METHODS

### A. Two‐dimensional dosimetry verification with film and ion chamber

The 2D dosimetry verification was performed with EBT2 GAFCHROMIC film (International Specialty Products, Wayne, NJ) for gamma evaluation. Farmer ion chamber was used for absolute point dose measurement. Because the 2–3 arcs/fields within the plan for each region were optimized simultaneously by the treatment planning system (TPS), the homogeneous dose distribution was achieved among the arcs. However, there were concerns regarding hot/cold spots existing in the junction area between the arcs belonging to the separate neighboring plans. Therefore, attention was given to hot/cold spots in the junction areas between the neighboring plans during dose verification.

**Figure 1:**
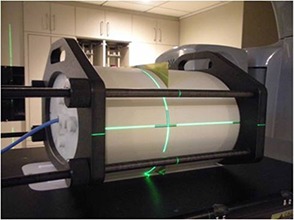
The setup of EBT2 GAF CHROMIC film and Farmer ion chamber with a homogeneous IMRT phantom for 2D dosimetry verification.

For each patient, an H&N and chest junction (H+C) phantom verification plan that only includes the most inferior arc/field of H&N plan and the most superior field of chest plan was generated in TPS. A chest and pelvis junction (C+P) verification plan was generated in the same fashion for each patient. The phantom used in this study was an IMRT phantom (CIRS Model 002H5, Computerized Imaging Reference Systems, Norfolk, VA). For both H+C and C+P plans, the plan dose at the film location and point dose at ion chamber location were calculated in the TPS and exported to be compared to the measurements. The plans were delivered on Varian 23 iX Clinac linear accelerator equipped with RapidArc (Varian Medical Systems, Palo Alto, CA). The setup of the film and ion chamber with the phantom is shown is Fig. 1. Since each junction in the verification plan was composed of 2 arc/fields with different isocenters, the EBT2 film and ion chamber were exposed to one arc/field first. Then the IMRT phantom was shifted to the second isocenter. And the film and chamber were exposed again. The films were digitized with the Epson Expression 10000XL flatbed scanner (US Epson, Long Beach, CA) and then carefully registered to the TPS output planar dose and analyzed in RIT software (Radiological Imaging Technology, Colorado Springs, CO). The gamma evaluation criteria were set as 5% absolute point dose difference and 3 mm of distance to agreement (DTA).

### B. Three‐dimensional dosimetry verification with EPID images and dose reconstruction

We conducted 3D dose verification with EPID images and a commercial dose reconstruction software, Dosimetry Check (Math Resolutions, LLC, Columbia, MD).^(^ ^16^ ^)^ In this verification, the treatment plan was copied as a QA plan and delivered. The portal images were acquired with Varian S‐1000 EPID (Varian Medical Systems) during a cine mode at 105 cm distance. A total of 64 EPID images were acquired for each gantry rotation. Considering the sampling nature of the cine acquisition mode, the same phantom QA plans was also delivered in an EPID integrate mode for total dose calibration for each arc/field. A 10×10 reference image was acquired weekly in an integrate mode with known dose used for final dose calibration. The computed tomography (CT) images with the structure set including body outlines, 3D dose matrix from TPS, and EPID files with beam geometry including gantry angles were transferred to Dosimetry Check program via DICOM RT format to reconstruct the volumetric dose and compare to the plan. For statistic gamma evaluation, we used body structure as a volume of interest. The same gamma evaluation criteria were used as in the 2D verification.

The Dosimetry Check uses the EPID images of the treatment fields to reconstruct the dose distribution in the CT planning model of the patient. As detailed by Renner,^(^ ^16^ ^)^ since the dose calculation algorithm is different from that used by the TPS, this provides an independent check of planning accuracy, as well as treatment delivery. Point doses, 3D dose distributions, gamma evaluations, and dose‐volume histogram (DVH) statistics can be compared within Dosimetry Check.

## III. RESULTS

**Figure 2:**
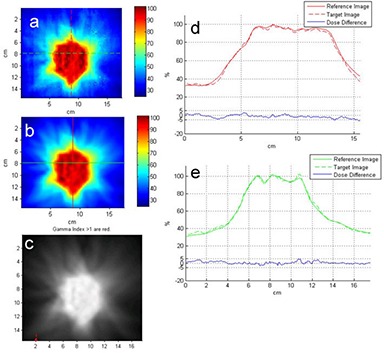
The comparison of the film measurement to the plan dose in the junction area of head and neck and chest regions in the an adult patient: (a) color‐coded film measurement (target image); (b) plan dose (reference image); (c) gamma evaluation (gamma indices > 1 are red); (d) comparison of the plan and measured dose profiles along the vertical line marked in (a) and (b); (e) comparison of the plan and measured dose profiles along the horizontal line marked in (a) and (b).

**Figure 3:**
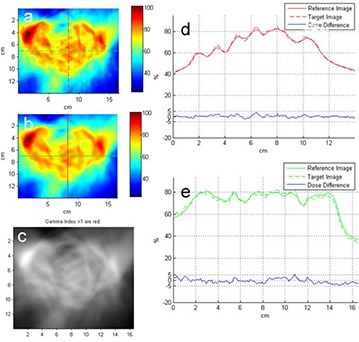
Comparison of the film measurement to the plan dose in the junction area of chest and pelvis regions in the same patient as shown in Fig. 2: (a) color‐coded film measurement (target image); (b) plan dose (reference image); (c) gamma evaluation (gamma indices > 1 are red); (d) comparison of the plan and measured dose profiles along the vertical line marked in (a) and (b); (e) comparison of the plan and measured dose profiles along the horizontal line marked in (a) and (b).

**Figure 4:**
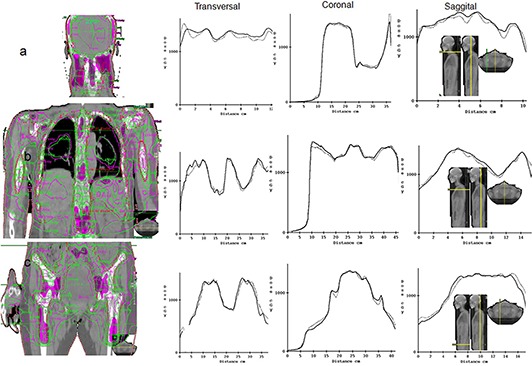
Comparison of the Dosimetry Check reconstructed isodose (magenta) to plan dose (green) in a patient, as shown in the coronal view. The head and neck plan (a) through the region's specific calculation points defined by the three cutoff planes indicated by yellow lines on the computed tomography (CT) scout images at right. Either the plan or Dosimetry Check dose may be tinted. The comparison of the reconstructed (solid) to plan (dotted) dose profile across transversal, coronal, and sagittal planes at the calculation point are shown. The isodose points for the chest and pelvic plans are shown in (b) and (c), respectively.

In all the TMI plans, adequate target dose coverage and reduced OARs dose were observed; more than 90% of PTV was covered by 100% of prescribed dose. Table 1 shows the absolute point dose difference between ion chamber measurements and plan, and gamma evaluation from 2D dosimetry with EBT2 GAFCHROMIC film in the junction planes in individual patient. The junction areas show overall average of 98.2% gamma passing rate and 3.9% absolute dose difference. Table 2 show the absolute point dose differences at isocenters between Dosimetry Check dose reconstruction and plans, as well as results of 3D gamma evaluation in the individual plan. The volumetric portal dosimetry shows overall average of 90.7% gamma passing rate and 2.4% absolute dose difference. The average absolute dose difference at the isocenter between reconstructed and plan dose is similar to the difference between chamber measurement and plan dose. Figures 2 and 3 demonstrate the good agreements between the film measurement and plan dose in the junction area of H&N and chest regions, and of chest and pelvis regions, respectively, in patient #3. The same extent of agreement was observed throughout this study. Figure 4 compares the Dosimetry Check reconstructed isodose (magenta) to plan dose (green) in the coronal view of the H&N, chest, and pelvic plans through the individual region's specific calculation points in patient #2. The comparison of the reconstructed dose (solid) to plan dose (dotted) profile across the transversal, coronal, and sagittal planes at the calculation point are also shown. Either the plan dose may be tinted in green, or the Dosimetry Check dose may be tinted in magenta for a particular isodose value, for ease of comparison. We observed that in low‐dose gradient area, the reconstructed dose lines were separated from plan dose lines by relative long distance, which suggests that such separation would only represent a small inconsequential disagreement in dose and recommends gamma evaluation as a better evaluation tool. It is consistent with previous publication of Dosimetry Check algorithm.^(^ ^16^ ^)^ Table 2 shows the absolute point dose difference at isocenters and gamma evaluation using volumetric dose reconstruction with EPID images in the plan region in the individual patients and overall site specific averages.

**Table 1:** Average absolute point dose difference from ion chamber point measurements and gamma evaluation (5% dose difference and 3 mm distance‐to‐agreement criteria) from 2D dosimetry with film in the junction area.

		*Patient #1*	*Patient #2*	*Patient #3*	*Average*
2D Gamma passing rate	H&N^a^/Chest	96.0%	97.2%	100.0%	97.7%
	Chest/Pelvis	100.0%	98.9%	96.8%	98.6%
Absolute point dose difference	H&N^a^/Chest	4.9%	6.2%	3.4%	4.8%
	Chest/Pelvis	0.5%	2.3%	6.3%	3.0%

a Head and neck

**Table 2:** Average absolute point dose difference and gamma evaluation (5% dose difference and 3 mm distance‐to‐agreement criteria) from volumetric portal dosimetry with electronic portal imaging device (EPID) in the plan region.

		*Patient #1*	*Patient #2*	*Patient #3*	*Average*
3D Gamma passing rate	H&N^a^	90.4%	96.4%	91.4%	92.7%
	Chest	90.7%	94.5%	87.8%	91.0%
	Pelvis	87.4%	95.2%	83.0%	88.5%
Absolute point dose difference	H&N^a^	0.3%	4.0%	3.9%	2.7%
	Chest	2.0%	3.5%	2.0%	2.5%
	Pelvis	2.3%	2.2%	2.2%	2.2%

a Head and neck

VMAT‐based TMI decreased the total MUs to the range of 3000–4000 versus 6000–7000 with conventional TBI. This shortened the beam‐on time to 1 minute for each arc/field at a dose rate of 600 cGy/min. We expect the treatment time can be much less than conventional TBI, even after considering the patient initial setup, image guidance before treatment, isocenter shifts, and possible midtreatment imaging for reassurance of the position.

## IV. DISCUSSION

This study demonstrated a dosimetry verification procedures involving both 2D and 3D for VMAT based TMI. Attention was also paid to the junction regions, which is particularly important to the type PTV in the case of TMI. Dosimetry verification for VMAT is relatively new compared to the more established fixed‐field IMRT by 2D arrays. Verifying the whole plan while the gantry is rotating is rather challenging. Considering this, we measured the VMAT‐based plan dose distributions using various dosimetric techniques and equipments: film and ion chamber measurements with an IMRT phantom and volumetric dose reconstructed from EPID images acquired during a cine mode. The point dosimetry allows for absolute dose distribution at individual points. Film dosimetry allows thorough validation and QA of relative dose distribution with higher spatial resolution compared to 2D ionization chamber array devices. The concerns of hot/cold spots on the junction area between the neighboring plans were addressed. The dosimetric verification of IMRT may require a dosimetry system with full capability in 3D, which is highly desirable when commissioning of a new IMRT technique, such as the study presented here. There have been efforts in developing gel‐based 3D dosimetry detectors relying on two main dose‐readout methods: magnetic resonance imaging (MRI) and optical CT. At present, those techniques are still in development with limited commercial support.^(^ ^17^ ^)^

In this study, we measured the volumetric dose with 3D dose reconstruction algorithms that relate the measured portal images to the dose delivered patient. Our institution has experience in utilizing commercial systems for IMRT dosimetry with established criteria for evaluation. The EPID quality assurance program is routinely validated against reference dosimeters of appropriate accuracy and resolution. Since the dose calculation algorithm is different from that used by the TPS, this provides an independent check of planning accuracy, as well as treatment delivery. We set the criteria as 5% absolute point dose difference and 3 mm of DTA, which is reasonable considering the geometric complexity of the target and its large volume. We observed better gamma passing rate in 2D verification compared to 3D volumetric verification. We did not conclude that the results from 3D verification were worse than 2D verification simply based on gamma passing rate, since previous studies have found a lack of statistical correlation between gamma passing rate in IMRT QA performance and dose errors in anatomic regions of interest, suggesting that the common acceptance criteria may have insufficient predictive power for per‐patient IMRT QA.^(^ ^18^ ^)^

The initial results from treatment planning and dosimetry verification are encouraging and warrant further investigation in clinical trials. The clinical implementation is challenging since the large treatment volume with consequence of multiple isocenters requires accurate patient positioning. A whole body immobilization system similar to the modular structure for setting up complex stereotactic body radiotherapy treatment may be useful in initial setting up patients and giving support during the treatment. The immobilization system should be indexed to reproduce the position on a daily basis. Studies of patient position reproducibility are needed. Daily image guidance will likely be required to verify the patient's alignment prior to treatment. Schultheiss et al.^(^ ^5^ ^)^ reportedly used two megavoltage CT scans (scan 1 – orbit to upper chest, scan 2 – top of the kidneys to the iliac crest) in their HT based IM‐TMI. The kilovoltage (kV) CBCT is an option widely available on linac systems. The current kV CBCT scan is limited to 15 cm in the scan length. Therefore, serial CBCT scans will be needed. Considering children are more sensitive to radiation, the ongoing low‐dose kV CBCT studies may be a solution. Previously we have developed three isocenter techniques on TMI planning for small children, which may be another solution with relatively simple patient setup.^(^ ^11^ ^)^

Several studies have reported a decrease in the relapse rate when increasing the total dose of TBI without increasing the pulmonary complications.^(^ ^19^ ^,^ ^20^ ^)^ Dose escalation may be performed in such a way that only the dose in the hematopoietically active bone marrow sites are escalated to a higher value, using simultaneous boost technique. Our research group has developed techniques that incorporating functional image modalities (e.g., quantitative fat fraction magnetic resonance imaging and 18‐fluorodeoxy‐D‐glucose positron emission tomography) to identify and spare active bone marrow for patients with pelvic malignancies undergoing concurrent chemoradiotherapy.^(^ ^21^ ^)^ Functional imaging could be applied for dose‐escalated TMI using active bone marrow as a separate target.

## V. CONCLUSIONS

VMAT‐based TMI/TMLI is a novel radiotherapy technique that could reduce toxicity, conform dose to the target, and permit dose escalation. This approach allows modern functional image guidance to be implemented, and therefore has significant potential to improve clinical outcomes. Successful implementation of this technique requires optimized planning and image‐guidance techniques to account for the large treatment fields and to ensure optimal patient immobilization and positioning. Comprehensive dosimetry verification procedures are also needed to assure accuracy of delivered dose. Future work will be focused on patient setup, image‐guided positioning, and organ motion management.

## Supporting information

Supplementary Material

Supplementary Material
