# Supplementary figures and images for: Feasibility study on dosimetry verification of volumetric‐modulated arc therapy‐based total marrow irradiation
Source: J Appl Clin Med Phys. 2013 Mar 4;14(2):15–23. doi: 10.1120/jacmp.v14i2.3852 (PMC5714362; doi:10.1120/jacmp.v14i2.3852)

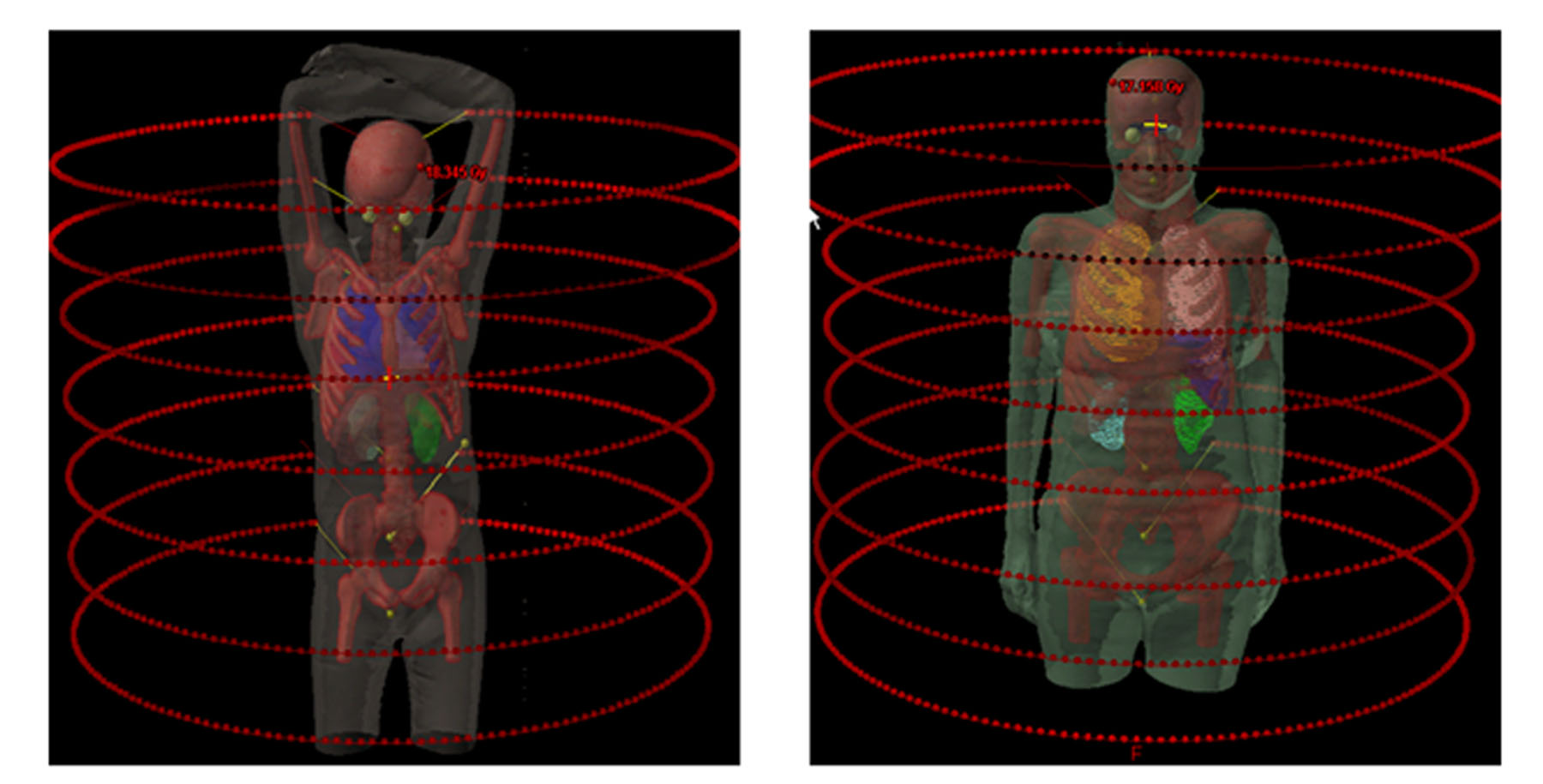

Supplement: Supplementary file 1 — Supplementary Material [file ACM2-14-015-s001.jpg]

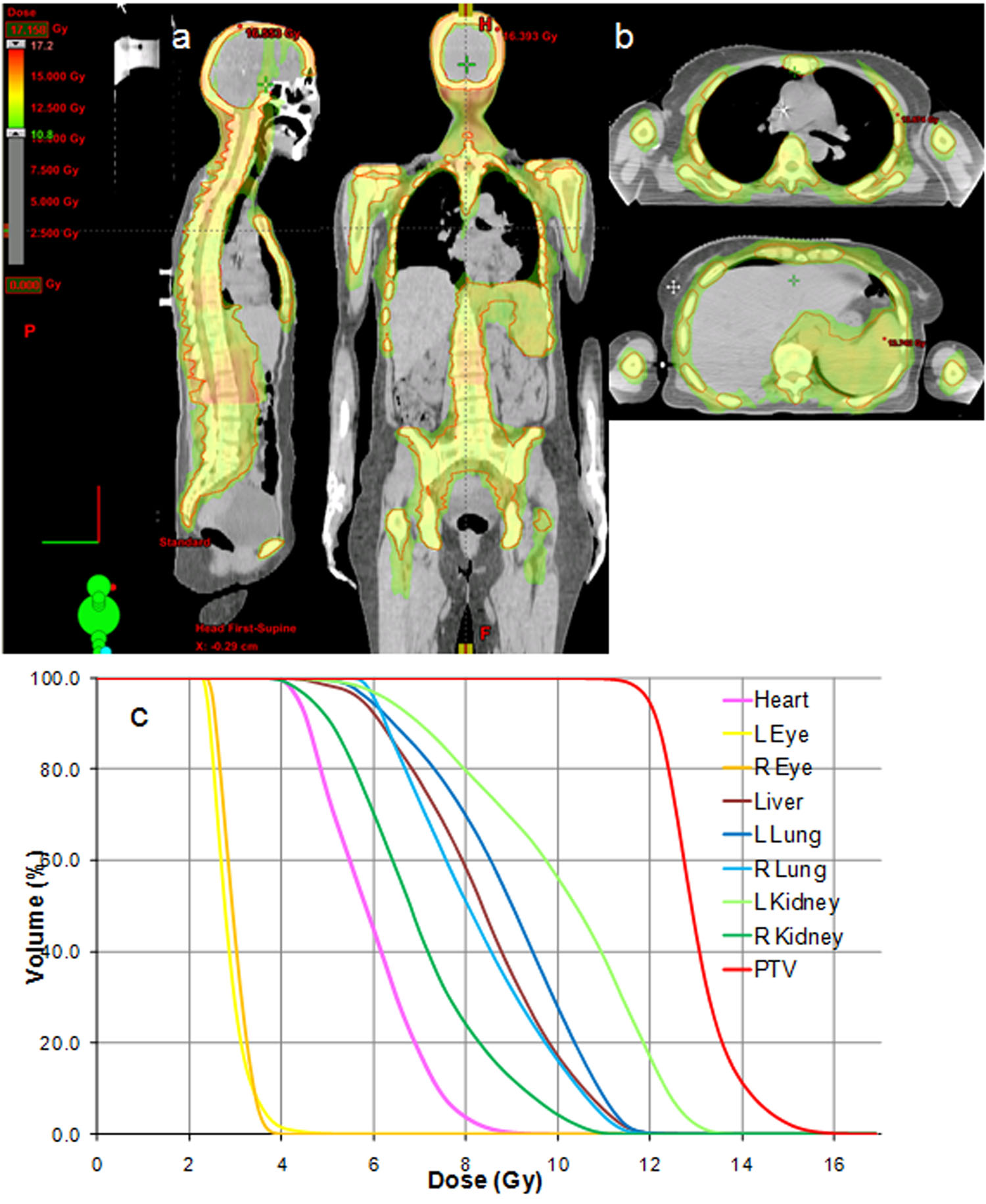

Supplement: Supplementary file 2 — Supplementary Material [file ACM2-14-015-s002.jpg]
